# Supplementary material for: Stable heteroplasmy at the single-cell level is facilitated by intercellular exchange of mtDNA
Source: Nucleic Acids Res. 2015 Feb 4;43(4):2177–87. doi: 10.1093/nar/gkv052 (PMC4344500; doi:10.1093/nar/gkv052)
Supplement: SUPPLEMENTARY DATA [file supp_43_4_2177__index.html]

Stable heteroplasmy at the single-cell level is facilitated by intercellular exchange of mtDNA — Stable heteroplasmy at the single-cell level is facilitated by intercellular exchange of mtDNA — Stable heteroplasmy at the single-cell level is facilitated by intercellular exchange of mtDNA — SUPPLEMENTARY DATA 

# Stable heteroplasmy at the single-cell level is facilitated by intercellular exchange of mtDNA

## SUPPLEMENTARY DATA

**Files in this Data Supplement:**

- SUPPLEMENTARY DATA
