# Supplementary material for: Stable heteroplasmy at the single-cell level is facilitated by intercellular exchange of mtDNA
Source: Nucleic Acids Res. 2015 Feb 4;43(4):2177–87. doi: 10.1093/nar/gkv052 (PMC4344500; doi:10.1093/nar/gkv052)
Supplement: SUPPLEMENTARY DATA [file supp_gkv052_nar-03262-n-2014-File002.pdf]

SUPPLEMENTARY MATERIAL

This accompanies the paper titled *Stable heteroplasmy at the single cell level is facilitated by inter-cellular exchange of mtDNA* by Jayaprakash *et al.*

Comparison of Mseek to other techniques

We tested several kits on the market by PCR using a combination of nuclear DNA and mtDNA specific primers and followed it up with deep sequencing only if the PCR data looked promising. Approaches using SNP arrays (23andMe) don’t have the resolution offered by Mseek while PCR-based approaches (e.g. Life technologies) can be excluded for reasons discussed in the introduction. Most other kits on the market for mtDNA purification involve lysing cells to release the organelles either chemically or by use of a dounce followed by the use of one of the following technologies,

- Purification of mitochondrial DNA from organelles using Enzyme B mix from Abcam to clean up the DNases and other proteins (kits from Abcam, PromoKine and BioVision),
- Magentic capture of mitochondria using anti-TOM22 microBeads to target the translocase of the outer mitochondrial membrane 22 (TOM 22) (MACS from Miltenyi Biotec)
- Amplification from total DNA with mtDNA specific primers (REPLI-g from Qiagen),
- Extraction of an mtDNA-enriched fraction using Qiagen’s plasmid miniprep kit followed by bead purification using Agencourt AMPure XP system(1).

We first tested the mini-prep method of isolating pure mtDNA(1). Approximately 20 million cells (HEK 293T) were used for each isolation. The cells were loaded onto a spin mini prep column and isolation was performed according to the manufacturer’s protocol (QIAGEN spin mini prep catalog # 27115). The mtDNA which is similar to plasmid DNA (size and circular structure) was eluted in 100 μl of elution buffer. The mtDNA enriched fraction was later purified using the AMPure XP system. A 0.4 X proportion of beads by volume were added, collected on the magnetic stand and washed twice with 80% ethanol. Post washing, the mtDNA was resuspended in 25 ul 0.1 X TE buffer. On testing with PCR, nDNA bands were seen(**Fig. S5A**), consistent with the published data(1) showing that the product had about 78% nuclear DNA(1). In that study, a subsequent mtDNA-specific amplification yielded high purity mtDNA.

We also tested the Miltenyi biotech Mitochondria MidiMACS starting kit, human (catalog # 130-094-872), since it is used by many commercial kits. Mammalian cells ($\approx 10^7$) were lysed and mitochondria were magnetically labeled with Anti-Tom22 microbeads which bind to the translocase of the outer mitochondrial membrane 22 protein (TOM 22). The sample was then loaded onto the column placed in the MACS separator. After washing only magnetically labeled mitochondria are retained on the column. The column is detached from the separator and mitochondrial organelles are eluted. Mitochondrial DNA is further isolated from the organelles by isopropanol precipitation. Since nuclear DNA remained in the MACS-purified samples according to PCR(**Fig. S5B**), consistent with the published data(1), sequencing was not performed.

We compared Mseek to REPLI-g (Qiagen), using sequencing on mtDNA from a blood sample (in triplicate) to establish that Mseek exhibits the highest purity of mtDNA compared to kits currently available on the market(**Fig. S5C**). Mseek data from cell lines shows substantial reduction of nuclear DNA and uniform coverage across the length of mtDNA(**Fig. 1**).

REFERENCES

1. Quispe-Tintaya, W., White, R.R., Popov, V.N., Vijg, J., Maslov, A.Y.: Fast mitochondrial DNA isolation from mammalian cells for next-generation sequencing. BioTechniques **55**(3), 133–136 (2013)
2. Jukes, T.H., Osawa, S.: The genetic code in mitochondria and chloroplasts. Experientia **46**(11-12), 1117–1126 (1990). Accessed 2014-07-08
3. Schmitt, M.W., Kennedy, S.R., Salk, J.J., Fox, E.J., Hiatt, J.B., Loeb, L.A.: Detection of ultra-rare mutations by next-generation sequencing. Proceedings of the National Academy of Sciences **109**(36), 14508–14513 (2012). Accessed 2014-09-24

Table ST1. Primers specific to human nuclear DNA.

gene	forward primer	reverse primer	Product size
hSox2	TTTTGTCGGAGACGGAGAAG	CATGAGCGTCTTGGTTTTCC	119 bp
hKlf4	ACCCTGGGTCTTGAGGAAGT	AGGAAGGATGGGTAATTGGG	104 bp
hOct4	GTGGAGAGCAACTCCGATG	TTGATGTCCTGGGACTCCTC	113 bp
hMyc	AAGGACTATCCTGCTGCCAA	CCTCTTGACATTCTCCTCGG	120 bp
hGapdh	CTCTGCTCCTCCTGTTCGAC	AATCCGTTGACTCCGACCTT	345 bp
FMR1	GCTCAGCTCCGTTTCGGTTTCACTTCCGGT	AGCCCCGCACTTCCACCACCAGCTCCTCCA	281 bp
AR	TCCAGAATCTGTTCCAGAGCGTGC	GCTGTGAAGGTTGCTGTTCCTCAT	288 bp
18S	GCAATTATTCCCCATGAACG	GGGACTTAATCAACGCAAGC	68 bp

Figure S1. Organization of mtDNA. 13 protein-coding genes, 22 tRNA genes and 2 rRNA genes are encoded by a single circular nucleic acid and transcribed from three promoters: LSP (inner circle of genes - strand), HSP1 (outer circle of gene, + strand) and HSP2 (16S rRNA) on the D-loop, which is non-coding but critical for replication and transcription. The three polycistronic transcripts are processed by enzymatic excision of the tRNAs. There are a few small gaps (<30 nt) in annotation, and a 45nt overlap between ATP6 and ATP8 which might have roles in replication. The mitochondrial genetic code differs from the nuclear code. In mammalian mitochondria, *ATA* codes for Methionine instead of Isoleucine, *TGA* codes for Tryptophan instead of the stop codon, and *AGA*, *AGG* code for stop codons instead of Arginine hinting at a bacterial origin(2).

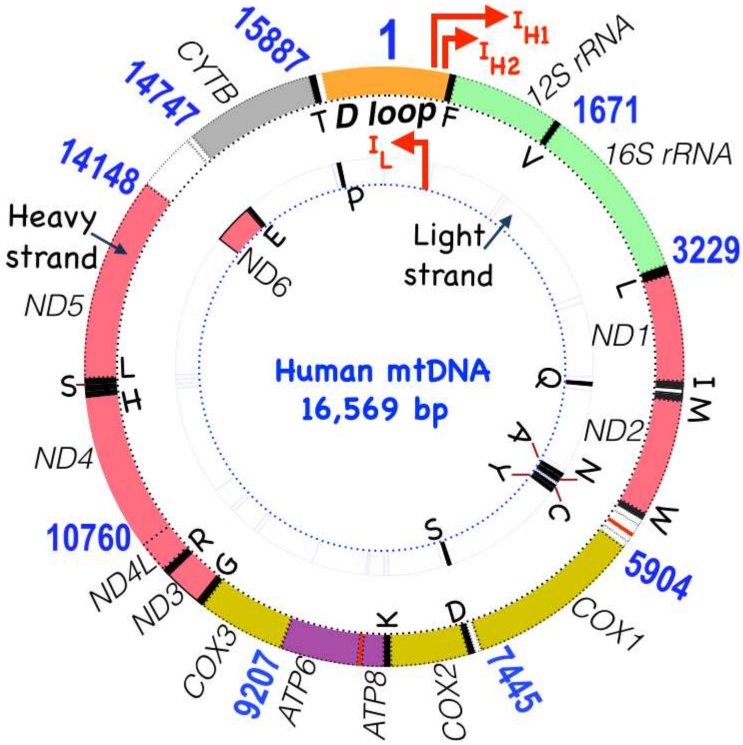

Table ST2. Primers specific to human mtDNA. The last row is the primer pair for amplifying the whole mtDNA using long-range PCR.

names	forward primer	reverse primer	Product size (bp)
mt pair.1	CTATCACCCTATTAACCACTCA	TTCGCCTGTAATATTGAACGTA	179
mt pair.21	AATCCAAGCCTACGTTTTCACA	AGTATGAGGAGCGTTATGGAGT	190
mt pair.31	TAACCCAAGTCAATAGAAGCCG	TAAGTTTCATAAGGGCTATCGT	497
mt pair.263	ATCTATTACTCTCATCGCTACC	TGTGATGCTAGGGTAGAATCCG	373
mt pair.19	AATCGAGTAGTACTCCCGATTG	TTCTAGGACGATGGGCATGAAA	218
mt pair.27	TCATCCCTGTAGCATTGTTCGT	TCGGGCGTATCATCAACTGATG	217
long-range	TCGGGCGTATCATCAACTGATG	CAGATGCCAACACAGCAGCCAT	16,568

Table ST3. Transitions versus Transversions for data from IMR90, WI-38 and MDA-MB-157. Of a total of 84 single base mutations there are only 6 transversions, the rest are transitions. The row labels are the original reference base, the column labels are the variant bases, so G to A changes happened 15 times. Most mutations are transitions, suggesting that the mutations do not arise from oxidative stress, instead, most are likely due to errors introduced by polymerase-γ activity(3).

	**a**	**c**	**g**	**t**
**A**	0	1	23	0
**C**	2	0	0	17
**G**	15	3	0	0
**T**	0	23	0	0

Figure S2. Distribution of Numts (Nuclear-mtDNA pseudogenes). The x-axis is the position along the mtDNA. The graph on the top shows the number of matches on the nuclear genome (hg38) of 36 nt tiles from the mtDNA. The graph in the bottom shows the changes in the mapping numbers for the tiles between hg38 and hg19, positive numbers are an increase in matches, while negative numbers are a decrease.

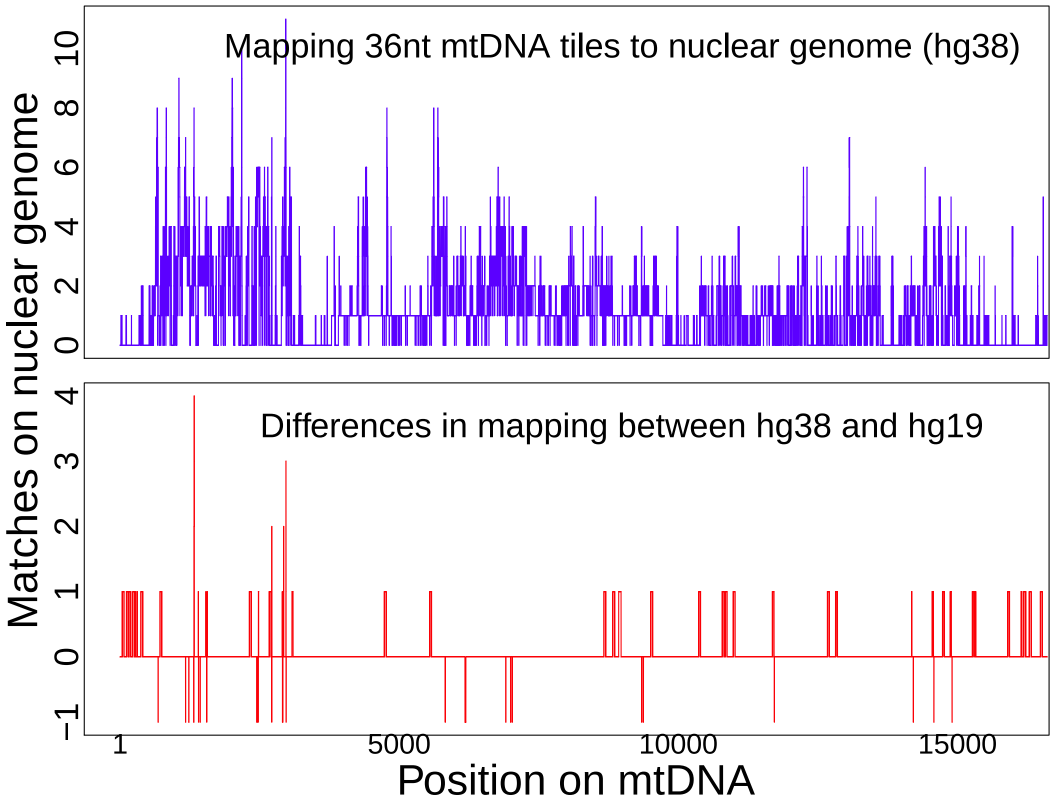

Figure S3. Distribution of Numts (Nuclear-mtDNA pseudogenes). The x-axis is the position along the mtDNA. The graph on the top shows the number of matches on the nuclear genome (hg38) of 100nt tiles from the mtDNA. The graph in the bottom shows the changes in the mapping numbers for the tiles between hg38 and hg19, positive numbers are an increase in matches, while negative numbers are a decrease. Even at 100nt, there are a number of Numts in the reference genome.

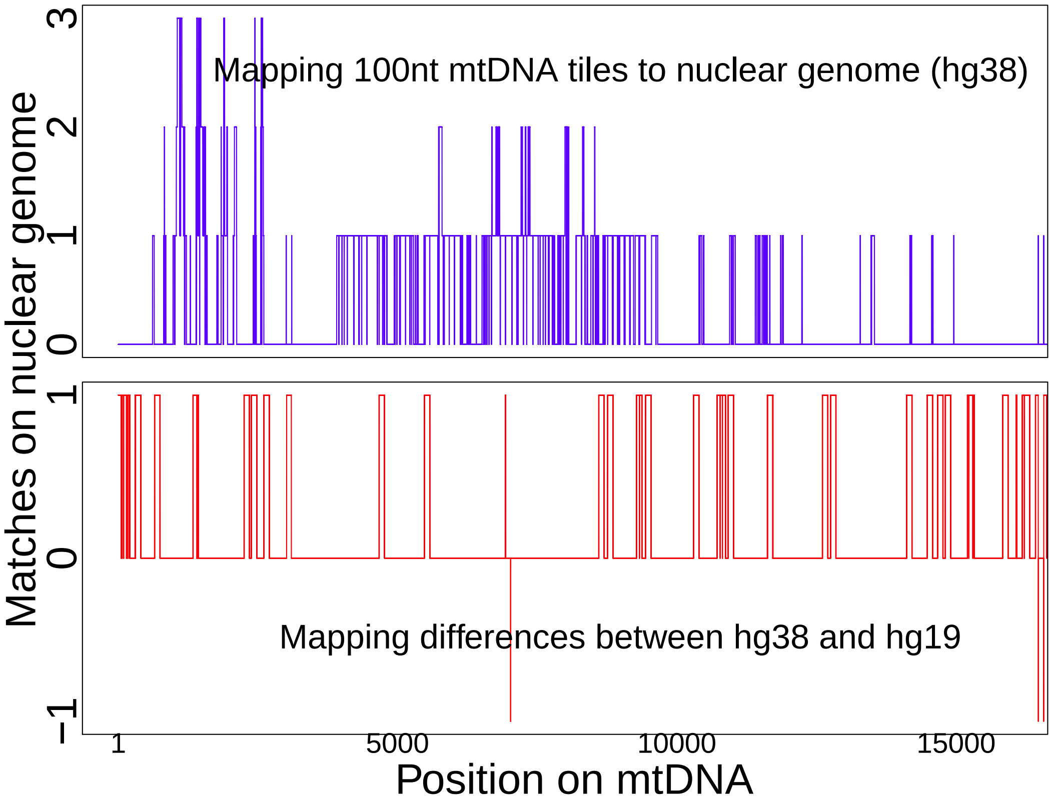

Figure S4. The Mseek protocol. The various steps of the protocol are shown here. Care must be taken in isolating total DNA, avoiding excessive cetrifugation for example, to avoid disrupting circular mtDNA. A sonicator (e.g. Covaris) is used to fragment DNA. The steps after fragmentation follow standard protocols for ChIP-seq.

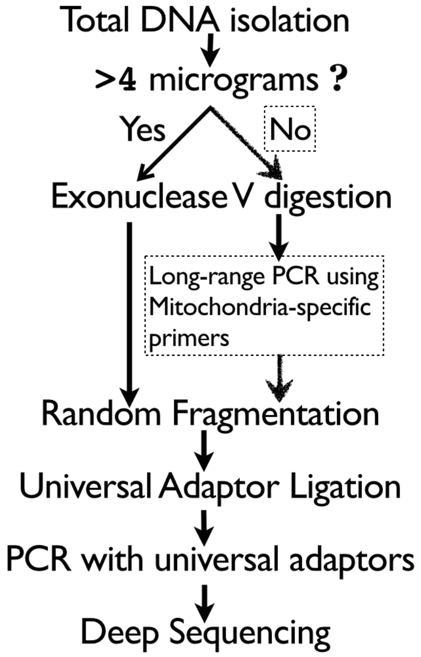

Figure S5. Mseek comparisons to other techniques. Plasmid mini-prep (panel A) and MACS (panel B) show a lack of purification of mtDNA based on PCR products run on a gel, thus they were not prepared for deep sequencing. Panel C shows sequencing data from REPLI-g (Qiagen) compared to Mseek, both of which show purification of mtDNA on the gel. **A** Plasmid mini prep followed by bead purification. PCR using primers specific to 18S(1), 28S(2), B-actin(3), mtDNA(4), mtDNA(5) show nDNA is present after treatment. **B** MACS. PCR using primers specific to 18S(1), 28S(2), B-actin(3), GAPDH(4), mtDNA(5), mtDNA(6) show that nDNA is present after prep.**C** Deep-sequencing data. Mseek (red bars) exhibits consistently better enrichment of mtDNA compared to REPLI-g (green bars) and untreated total DNA (blue bars). This comparison was done in triplicates, each using 8 μg of total DNA from the same blood sample. The purity of mtDNA from Mseek (80%) is lower compared to results presented in the text from cell lines, due to various factors in blood which requires further fine-tuning.

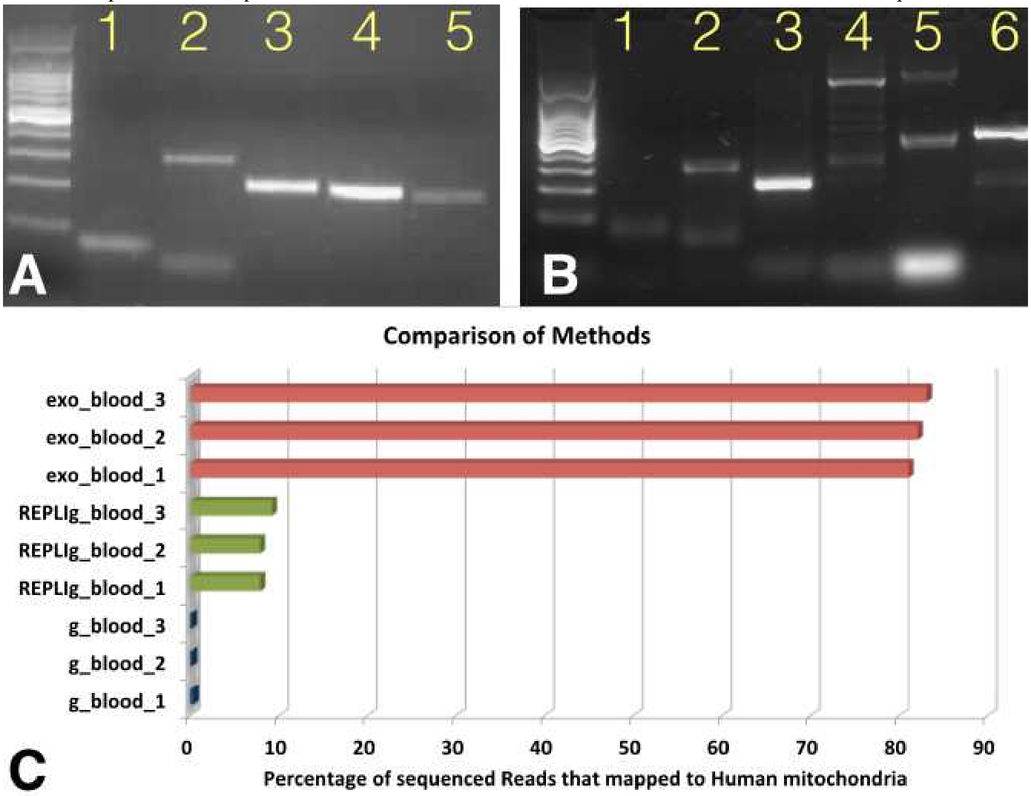

Table ST4. Co-culture of WI-38-GFP(fibroblast) cells with IMR90 (fibroblast) cells for 4 weeks. After the co-culturing, GFP negative cells were killed using a neomycin selection marker, which was incorporated with the GFP. The white rows are variants private to WI-38-GFP, gray rows show variant or reference alleles private to IMR90 that appear in WI-38-GFP after co-culture, blue rows are variants common to IMR90 and WI-38 while the green rows are variants private to IMR90 with minimal or no transfer. The frequency (*freq*) ranges from 0-1 and the coverage (*cov*) is the number of reads at the variant. For example in row 11, *G* at position 3196 is mutated to an *A* in WI-38, while the IMR90 has the *G* at that position. After co-culturing, at position 3196 in the WI-38 cells a *G* occurs in 52% (0.52) of the reads.

Variant	gene	AA	IMR90 control		WI-38-GFP control		WI-38-GFP mixed with IMR90		flank
			freq	cov	freq	cov	freq	cov	
C194t	D-loop		0.67	31	0	140	0.05	107	CGAACATA[C|t]TTACTAAA
T195c	D-loop		0.76	30	0	143	0.07	104	GAACATAC[T|c]TACTAAAG
G207a	D-loop		0.88	63	0	133	0.07	104	TAAAGTGT[G|a]TTAATTAA
A189g	D-loop		0.93	63	0	145	0.10	108	ACAGGCGA[A|g]CATACTTA
T204c	D-loop		0.90	62	0	132	0.10	104	TACTAAAG[T|c]GTGTTAAT
C514del(CA)	D-loop		0.32	306	0	118	0.10	109	CTACCCAG[C|del(CA)]ACACACAC
A519del(AC)	D-loop		0.17	317	0	123	0.12	111	CAGCACAC[A|del(AC)]CACACCGC
G709a	s-rRNA		0.94	226	0	111	0.27	113	GCATCCCC[G|a]TTCCAGTG
T1243c	s-rRNA		0.97	298	0	126	0.27	118	TCACCACC[T|c]CTTGCTCA
T1406c	s-rRNA		0.96	179	0	110	0.25	93	TATGAAAC[T|c]TAAGGGTC
G3196a	l-rRNA		0	507	0.94	56	0.48	94	TCAACTTA[G|a]TATTATAC
T3197c	l-rRNA		0	510	0.94	56	0.5	94	CAACTTAG[T|c]ATTATACC
A3505g	ND1	T->A	0.95	318	0	153	0.24	132	CCACATCT[A|g]CCATCACC
G5046a	ND2	V->I	0.97	326	0	120	0.288	125	TAATAGCA[G|a]TTCTACCG
G5460a	ND2	A->T	0.90	407	0	113	0.31	123	CACTCATC[G|a]CCCTTACC
C6434t	COX1	T->T	0	494	0.91	83	0.61	107	GCCATAAC[C|t]CAATACCA
G8251a	COX2	G->G	0.94	247	0	107	0.29	112	GAAATAGG[G|a]CCCGTATT
G8994a	ATP6	L->L	0.96	269	0	100	0.30	115	ATAGCCCT[G|a]GCCGTACG
G9477a	COX3	V->I	0	469	1	58	0.52	97	CCTCAGAA[G|a]TTTTTTTC
A11467g	ND4	L->L	0	516	0.95	60	0.57	97	GTACTCTT[A|g]AAACTAGG
C11674t	ND4	T->T	0.92	267	0	98	0.25	108	ATCCAAAC[C|t]CCCTGAAG
A11947g	ND4	T->T	0.96	319	0	135	0.25	127	CTACTTAC[A|g]GGACTCAA
A12308g	tRNA-Leu		0	591	0.97	79	0.57	102	GGCCCCAA[A|g]AATTTTGG
G12372a	ND5	L->L	0	683	0.90	95	0.56	112	CTAACCCT[G|a]ACTTCCCT
T12414c	ND5	P->P	0.94	353	0	140	0.28	133	GTTAACCC[T|c]AACAAAAA
C12705t	ND5	I->I	0.98	304	0	114	0.27	121	CTACTCAT[C|t]TTCCTAAT
A13263g	ND5	Q->Q	0.97	253	0	110	0.30	112	TCAAGTCA[A|g]CTAGGACT
T13617c	ND5	I->I	0	561	0.97	72	0.52	100	CGAATAAT[T|c]CTTCTCAC
A13827g	ND5	G->G	0	533	0.95	83	0.54	111	TTCCTAGG[A|g]CTTCTAAC
G13928c	ND5	S->T	0	593	0.95	70	0.53	103	CTACCCTA[G|c]CATCACAC
A14793g	CYTB	H->R	0	474	0.93	45	0.43	81	AATTAACC[A|g]CTCATTCA
T15784c	CYTB	P->P	0.97	339	0	95	0.29	110	AGCTACCC[T|c]TTTACCAT
G15884c	CYTB	A->P	0.95	309	0	122	0.29	117	TCAAATGG[G|c]CCTGTCCT
C16114a	D-loop		0	662	0.92	79	0.53	107	GCCAGCCA[C|a]CATGAATA
C16192t	D-loop		0	601	0.92	77	0.55	100	CCCCCTCC[C|t]CATGCTTA
C16223t	D-loop		0.97	283	0	110	0.27	100	AATCAACC[C|t]TCAACTAT
C16256t	D-loop		0	520	0.95	47	0.40	72	CTCCAAAG[C|t]CACCCCTC
C16270t	D-loop		0	480	0.93	16	0.15	52	CTCACCCA[C|t]TAGGATAC
C16292t	D-loop		0.97	338	0	40	0.51	64	AACCTACC[C|t]ACCCTTAA
C16294t	D-loop		0	382	1	40	0.43	69	CCTACCCA[C|t]CCTTAACA
T16519c	D-loop		0.98	258	0	88	0.34	108	CTTCAGGG[T|c]CATAAAGC
G16526a	D-loop		0	316	0.94	75	0.54	102	GTCATAAA[G|a]CCTAAATA
A464c	D-loop		0	715	0.11	191	0.13	127	CCCCTCCC[A|c]CTCCCATA
A2784g	l-rRNA		0	421	0.25	127	0.35	108	GGTCCTAA[A|g]CTACCAAA
C315Cc	D-loop		0	322	0.08	94	0.08	74	CCCTCCCC[C|Cc]GCTTCTGG
A73g	D-loop		0.97	405	1	78	1	73	TGGGGGGT[A|g]TGCACGCG
A263g	D-loop		0.97	335	0.98	63	1	58	GCACAGCC[A|g]CTTTCCAC
C309Cct	D-loop		0.28	280	0.54	81	0.32	64	AACCCCCC[C|Cct]TCCCCCGC
T310c	D-loop		0.77	272	0.87	79	0.90	62	ACCCCCCC[T|c]CCCCCGCT
G316c	D-loop		0.11	303	0.18	95	0.10	69	CCTCCCCC[G|c]CTTCTGGC
A750g	s-rRNA		0.97	202	0.98	68	1	63	TCAAAAGG[A|g]ACAAGCAT
C1290a	s-rRNA		0.07	571	0.08	126	0.05	102	GATGAAGG[C|a]TACAAAGT
A1438g	s-rRNA		0.98	170	1	58	1	50	AGTAAACT[A|g]AGAGTAGA
A2706g	l-rRNA		0.976	250	1	69	1	55	GCGGGCAT[A|g]ACACAGCA
A2838g	l-rRNA		0.21	507	0.192	125	0.15	97	AGAACCCA[A|g]CCTCCGAG
A3107del(A)	l-rRNA		0.97	201	1	46	1	42	CTATCTAC[A|del(A)]TTCAAATT
A4769g	ND2	M->M	0.97	233	1	60	1	50	ATCATAAT[A|g]GCTATAGC
T5734c	tRNA-Asn-tRNA-Cys		0.09	731	0.13	174	0.17	116	TCTACTTC[T|c]CCCGCCGC
G5746a	tRNA-Asn-tRNA-Cys		0.09	650	0.14	171	0.08	118	GCCGCCGG[G|a]AAAAAAGG
C7028t	COX1	A->A	0.97	284	1	73	0.96	53	GTTGTAGC[C|t]CACTTCCA
A8860g	ATP6	T->A	0.98	296	1	81	1	57	GAGCGGGC[A|g]CAGTGATT
T10941c	ND4	L->P	0.14	1685	0.11	384	0.15	324	CGACCCCC[T|c]AACAACCC
A10946c	ND4	T->P	0.11	1638	0.08	357	0.09	296	CCCTAACA[A|c]CCCCCCTC
T10953c	ND4	L->P	0.26	1395	0.24	297	0.26	225	AACCCCCC[T|c]CCTAATAC
T10956c	ND4	L->P	0.20	1302	0.17	281	0.22	208	CCCCCTCC[T|c]AATACTAA
G11719a	ND4	G->G	0.96	235	0.96	62	0.98	65	GCCCACGG[G|a]CTTACATC
C14766t	CYTB	T->I	0.96	464	0.98	56	0.98	52	ACGCAAAA[C|t]TAACCCCC
A15326g	CYTB	T->A	0.98	309	1	83	0.98	66	CCCTAGCA[A|g]CACTCCAC

Table ST5. Co-culture of MDA-MB-157 cells with HCC1806 cells for 4 weeks. GFP-labeled MDA-MB-157 cells made up about 10% of the mixture, which was passaged 25 times before the component cells were separated using FACS. The mtDNA was sequenced to identify variants in the GFP cells. The individual cell lines were previously sequenced to identify variants private to each cell line. The private_to column identifies the cell-lines that exhibit the variant. Rows highlighted in gray are cases where a variant or reference allele unique to HCC1806 has been identified in MDA-MB-157 cells after co-culture. The light green rows are variants private to HCC1806 that did not transfer into MDA-MB-157. Rows highlighted in blue show variants common to both cell lines. The frequency (*freq*) ranges from 0-1 and the coverage (*cov*) is the number of reads at the variant. For example, at position 3796 (row 6), the *A* from the reference mtDNA genome is mutated to a *T* only in HCC1806, MDA-MB-157 cultured with HCC1806 exhibits an *A* at 3796 in 14% (0.14) of the reads.

<table>
<tr><th rowspan="3">variant</th><th rowspan="3">gene</th><th rowspan="3">AA</th><th rowspan="3">private_to</th><th colspan="2">HCC1806 control</th><th colspan="2">MDA-MB-157 from HCC1806</th><th rowspan="3">flank</th></tr>
<tr></tr>
<tr><th>freq</th><th>cov</th><th>freq</th><th>cov</th></tr>
<tr><td>G3666A</td><td>ND1</td><td>G120G</td><td>HCC1806</td><td>0.93</td><td>33</td><td>0</td><td>25</td><td>TCTGATCAGG[G|a]TGAGCATCAA</td></tr>
<tr><td>A9545G</td><td>COX3</td><td>G113G</td><td>HCC1806</td><td>0.93</td><td>15</td><td>0</td><td>13</td><td>CCCAATTAGG[A|g]GGGCACTGGC</td></tr>
<tr><td>A12810G</td><td>ND5</td><td>W158W</td><td>HCC1806</td><td>0.92</td><td>25</td><td>0</td><td>20</td><td>AATCCTATAC[A|g]ACCGTATCGG</td></tr>
<tr><td>C14911T</td><td>CYTB</td><td>Y55Y</td><td>HCC1806</td><td>0.96</td><td>29</td><td>0</td><td>39</td><td>CTATATTACG[C|t]ATCATTTCTC</td></tr>
<tr><td>C16187T</td><td>D-loop</td><td></td><td>HCC1806</td><td>1</td><td>9</td><td>0</td><td>31</td><td>ATCAAAACCC[C|t]CTCCCCATGC</td></tr>
<tr><td>A3796T</td><td>ND1</td><td>T163S</td><td>HCC1806</td><td>0.88</td><td>9</td><td>0.14</td><td>21</td><td>TAACCTCTCC[A|t]CCCTTATCAC</td></tr>
<tr><td>A4104G</td><td>ND1</td><td>L266L</td><td>HCC1806</td><td>0.9</td><td>21</td><td>0.06</td><td>31</td><td>TTCTAACCTC[A|g]CTGTTCTTAT</td></tr>
<tr><td>T7861C</td><td>COX2</td><td>D92D</td><td>HCC1806</td><td>0.96</td><td>29</td><td>0.05</td><td>39</td><td>CGGACTAATC[T|c]TCAACTCCTA</td></tr>
<tr><td>G9064A</td><td>ATP6</td><td>A179T</td><td>HCC1806</td><td>1</td><td>10</td><td>0.16</td><td>24</td><td>CCATTAACCT[G|a]CCCTCTACAC</td></tr>
<tr><td>A9072G</td><td>ATP6</td><td>S182S</td><td>HCC1806</td><td>0.88</td><td>9</td><td>0.18</td><td>22</td><td>CTTCCCTCTA[A|g]ACTTATCATC</td></tr>
<tr><td>G10688A</td><td>ND4L</td><td>V73V</td><td>HCC1806</td><td>0.96</td><td>26</td><td>0.1</td><td>19</td><td>ACCTGACTCC[G|a]ACCCCTCACA</td></tr>
<tr><td>C7819A</td><td>COX2</td><td>L78L</td><td>MDA-MB-157</td><td>0</td><td>35</td><td>0.76</td><td>43</td><td>CGCATCCTTT[C|a]CATAACAGAC</td></tr>
<tr><td>C8932T</td><td>ATP6</td><td>P135S</td><td>MDA-MB-157</td><td>0</td><td>25</td><td>0.9</td><td>21</td><td>CCATACTAGT[C|t]ATTATCGAAA</td></tr>
<tr><td>T13602C</td><td>ND5</td><td>Y422Y</td><td>MDA-MB-157</td><td>0</td><td>22</td><td>0.45</td><td>24</td><td>CAAGCGCCTA[T|c]AGCACTCGAA</td></tr>
<tr><td>T15514C</td><td>CYTB</td><td>Y256Y</td><td>MDA-MB-157</td><td>0</td><td>12</td><td>0.89</td><td>28</td><td>CAGACAATTA[T|c]ACCCTAGCCA</td></tr>
<tr><td>T16209C</td><td>D-loop</td><td></td><td>MDA-MB-157</td><td>0</td><td>17</td><td>0.92</td><td>26</td><td>TACAAGCAAG[T|c]ACAGCAATCA</td></tr>
<tr><td>C16292T</td><td>D-loop</td><td></td><td>MDA-MB-157</td><td>0</td><td>15</td><td>0.76</td><td>21</td><td>CAAACCTACC[C|t]ACCCTTAACA</td></tr>
<tr><td>C16295T</td><td>D-loop</td><td></td><td>MDA-MB-157</td><td>0</td><td>14</td><td>0.72</td><td>22</td><td>ACCTACCCAC[C|t]CTTAACAGTA</td></tr>
<tr><td>T9540C</td><td>COX3</td><td>L111L</td><td>Both</td><td>0.86</td><td>15</td><td>1</td><td>11</td><td>TACCCCCCAA[T|c]TAGGAGGGCA</td></tr>
<tr><td>C16223T</td><td>D-loop</td><td></td><td>Both</td><td>0.95</td><td>20</td><td>0.96</td><td>31</td><td>GCAATCAACC[C|t]TCAACTATCA</td></tr>
<tr><td>T16311C</td><td>D-loop</td><td></td><td>Both</td><td>0.75</td><td>12</td><td>0.8</td><td>20</td><td>CAGTACATAG[T|c]ACATAAAGCC</td></tr>
</table>

Table ST6. Co-culture of MDA-MB-157-GFP(cancer) with IMR90(fibroblast). The white rows are variants private to MDA-MB-157-GFP, gray rows are variants or reference alleles unique to IMR90 that appear in MDA-MB-157-GFP after co-culture, blue rows are variants common to IMR90 and MDA-MB-157-GFP, and green rows are variants private to IMR90 that did not transfer. There were no transfers in this case. The frequency (*freq*) ranges from 0-1 and the coverage (*cov*) is the number of reads at the variant. For example, C194t refers to a reference *C* at position 194 mutated to a *T*, which occurs in 67% (0.67) of the reads in IMR90, but never (0%) in MDA-MB-157-GFP (before and after the mixing).

Variant	gene	AA	IMR90 control		MDA-MB-157-GFP control		MDA-MB-157-GFP mixed with IMR90		flank
			freq	cov	freq	cov	freq	cov	
C194t	D-loop		0.67	31	0	79	0	212	CGAACATA[C|t]TTACTAAA
T195c	D-loop		0.76	30	0	78	0	212	GAACATAC[T|c]TACTAAAG
T204c	D-loop		0.90	62	0	81	0	222	TACTAAAG[T|c]GTGTTAAT
G207a	D-loop		0.88	63	0	85	0	234	TAAAGTGT[G|a]TTAATTAA
C309Cct	D-loop		0.28	280	0	98	0	122	AACCCCCC[C|Cct]TCCCCCGC
C514del(CA)	D-loop		0.32	306	0	174	0	318	CTACCCAG[C|del(CA)]ACACACAC
A519del(AC)	D-loop		0.17	317	0	177	0	325	CAGCACAC[A|del(AC)]CACACCGC
G709a	s-rRNA		0.94	226	0	136	0	260	GCATCCCC[G|a]TTCCAGTG
T1243c	s-rRNA		0.97	298	0	147	0	313	TCACCACC[T|c]CTTGCTCA
T1406c	s-rRNA		0.96	179	0	126	0	240	TATGAAAC[T|c]TAAGGGTC
A3505g	ND1	T->A	0.95	318	0	189	0	395	CCACATCT[A|g]CCATCACC
G5046a	ND2	V->I	0.97	326	0	187	0	331	TAATAGCA[G|a]TTCTACCG
G5460a	ND2	A->T	0.90	407	0	171	0	340	CACTCATC[G|a]CCCTTACC
G8251a	COX2	G->G	0.94	247	0	166	0	279	GAAATAGG[G|a]CCCGTATT
C11674t	ND4	T->T	0.92	267	0	139	0	302	ATCCAAAC[C|t]CCCTGAAG
A11947g	ND4	T->T	0.96	319	0	200	0	397	CTACTTAC[A|g]GGACTCAA
T12414c	ND5	P->P	0.94	353	0	213	0	448	GTTAACCC[T|c]AACAAAAA
A13263g	ND5	Q->Q	0.97	253	0	180	0	336	TCAAGTCA[A|g]CTAGGACT
T15784c	CYTB	P->P	0.97	339	0	182	0	333	AGCTACCC[T|c]TTTACCAT
G15884c	CYTB	A->P	0.95	309	0	183	0	350	TCAAATGG[G|c]CCTGTCCT
C5601t	tRNA-Ala		0	503	0.94	92	0.94	153	CTGCAAAA[C|t]CCCACTCT
A6692del(A)	COX1	G->	0	594	0.17	202	0.16	280	TACTCCGG[A|del(A)]AAAAAAGA
A6696del(A)	COX1	K->	0	602	0.17	218	0.16	302	CCGGAAAA[A|del(A)]AAGAACCA
C7819a	COX2	L->L	0	535	0.92	99	0.95	167	ATCGCCCT[C|a]CCATCCCT
C8410t	ATP8	P->P	0	720	0.97	122	0.91	222	ATTACCCC[C|t]ATACTCCT
A8527g	ATP8	K->K	0	610	0.94	84	0.94	180	GAACCAAA[A|g]TGAACGAA
A8527g	ATP6	M->V	0	610	0.94	84	0.94	180	GAACCAAA[A|g]TGAACGAA
A8701g	ATP6	T->A	0	832	0.92	128	0.94	223	AAATGATA[A|g]CCATACAC
C8932t	ATP6	P->S	0	613	0.96	85	0.91	160	CACCTACA[C|t]CCCTTATC
T9540c	COX3	L->L	0	461	0.99	111	0.99	161	CCCCCCAA[T|c]TAGGAGGG
T9950c	COX3	V->V	0	482	0.90	93	0.86	154	GATGTGGT[T|c]TGACTATT
C10070t	ND3	A->A	0	492	0.95	94	0.92	121	AACTTCGC[C|t]TTAATTTT
A10398g	ND3	T->A	0	553	0.94	108	0.97	139	TAGACTGA[A|g]CCGAATTG
T10873c	ND4	P->P	0	674	0.98	113	0.96	212	ATCATCCC[T|c]CTACTATT
G11440a	ND4	G->G	0	518	0.92	122	0.88	189	ATCGCTGG[G|a]TCAATAGT
T13602c	ND5	Y->Y	0	565	0.38	180	0.31	316	AGCGCCTA[T|c]AGCACTCG
A14769g	CYTB	N->S	0	494	0.94	91	0.91	202	CAAAACTA[A|g]CCCCCTAA
G15301a	CYTB	L->L	0	512	0.95	48	0.87	94	TTCATCTT[G|a]CCCTTCAT
T15940del(T)	tRNA-Thr		0	668	0.32	107	0.37	174	TGAAAACC[T|del(T)]TTTTCCAA
T15944del(T)	tRNA-Thr		0	654	0.45	100	0.34	173	AACCTTTT[T|del(T)]CCAAGGAC
G16129a	D-loop		0	681	0.95	93	0.92	202	TATTGTAC[G|a]GTACCATA
T16209c	D-loop		0	456	0.95	60	0.91	111	CAAGCAAG[T|c]ACAGCAAT
C16295t	D-loop		0	393	0.94	38	0.93	65	CTACCCAC[C|t]CTTAACAG
T16311c	D-loop		0	457	0.98	51	0.91	80	GTACATAG[T|c]ACATAAAG
A200g	D-loop		0	75	0.96	58	0.95	173	TACTTACT[A|g]AAGTGTGT
T4218c	ND1	Y->Y	0	747	0.952	125	0.94	198	ATATGATA[T|c]GTCTCCAT
G4412a	tRNA-Met		0	489	0.35	175	0.32	272	TAAGGTCA[G|a]CTAAATAA
T1822c	l-rRNA		0	673	0.98	111	0.93	170	AAGCATAA[T|c]ATAGCAAG
T3396c	ND1	Y->Y	0	477	0.975	80	0.93	148	CTAGGCTA[T|c]ATACAACT
C311Ctcc	D-loop		0	294	0.19	105	0.12	138	CCCCCCCT[C|Ctcc]CCCCGCTT
A4769g	ND2	M->M	0.97	233	0.99	101	0.97	139	ATCATAAT[A|g]GCTATAGC
T5734c	tRNA-Asn-tRNA-Cys		0.09	731	0.11	230	0.13	436	TCTACTTC[T|c]CCCGCCGC
G5746a	tRNA-Asn-tRNA-Cys		0.09	650	0.09	232	0.11	381	GCCGCCGG[G|a]AAAAAAGG
C7028t	COX1	A->A	0.97	284	0.95	112	0.97	149	GTTGTAGC[C|t]CACTTCCA
A8860g	ATP6	T->A	0.98	296	1	93	0.95	176	GAGCGGGC[A|g]CAGTGATT
T10941c	ND4	L->P	0.14	1685	0.15	614	0.13	1069	CGACCCCC[T|c]AACAACCC
A10946c	ND4	T->P	0.11	1638	0.07	579	0.09	1027	CCCTAACA[A|c]CCCCCCTC
T10953c	ND4	L->P	0.26	1395	0.33	479	0.27	873	AACCCCCC[T|c]CCTAATAC
T10956c	ND4	L->P	0.20	1302	0.29	454	0.22	816	CCCCCTCC[T|c]AATACTAA
G11719a	ND4	G->G	0.96	235	0.98	94	0.95	141	GCCCACGG[G|a]CTTACATC
C12705t	ND5	I->I	0.98	304	0.99	116	0.96	160	CTACTCAT[C|t]TTCCTAAT
C14766t	CYTB	T->I	0.96	464	0.98	91	0.98	205	ACGCAAAA[C|t]TAACCCCC
A15326g	CYTB	T->A	0.98	309	0.98	58	0.98	92	CCCTAGCA[A|g]CACTCCAC
C16223t	D-loop		0.97	283	1	53	0.95	123	AATCAACC[C|t]TCAACTAT
C16292t	D-loop		0.97	338	1	37	0.93	64	AACCTACC[C|t]ACCCTTAA
T16519c	D-loop		0.98	258	1	87	0.96	160	CTTCAGGG[T|c]CATAAAGC
A73g	D-loop		0.97	405	0.97	181	0.95	286	TGGGGGGT[A|g]TGCACGCG
A189g	D-loop		0.93	63	0.93	62	0.94	169	ACAGGCGA[A|g]CATACTTA
A263g	D-loop		0.97	335	0.99	146	0.97	246	GCACAGCC[A|g]CTTTCCAC
T310c	D-loop		0.77	272	0.82	95	0.83	116	ACCCCCCC[T|c]CCCCCGCT
G316c	D-loop		0.11	303	0.05	115	0.06	147	CCTCCCCC[G|c]CTTCTGGC
A750g	s-rRNA		0.97	202	0.97	90	0.96	130	TCAAAAGG[A|g]ACAAGCAT
C1290a	s-rRNA		0.07	571	0.11	157	0.08	320	GATGAAGG[C|a]TACAAAGT
A1438g	s-rRNA		0.98	170	0.98	74	0.98	122	AGTAAACT[A|g]AGAGTAGA
A2706g	l-rRNA		0.976	250	0.96	92	0.96	149	GCGGGCAT[A|g]ACACAGCA
A2838g	l-rRNA		0.21	507	0.16	156	0.26	294	AGAACCCA[A|g]CCTCCGAG
A3107del(A)	l-rRNA		0.97	201	1	59	0.98	101	CTATCTAC[A|del(A)]TTCAAATT

Table ST7. Co-culture of U20S-GFP with A382 for 4 weeks. The column private_to identifies the cell-lines that exhibit the variant. The light green rows are variants private to A382 that did not transfer into U20S. The white rows are variants private to U20S. No transfers occured in this case. The frequency is *freq* (which ranges from 0-1) and the coverage *cov* is the number of reads covering the variant.

<table>
<tr><th>mut</th><th>gene</th><th>AA</th><th>private_to</th><th colspan="2">U20S-GFP from A382</th><th>flank</th></tr>
<tr><th></th><th></th><th></th><th></th><th>freq</th><th>cov</th><th></th></tr>
<tr><td>A8701G</td><td>ATP6</td><td>T58A</td><td>A382</td><td>0</td><td>121</td><td>ACAAATGATA[A|g]CCATACACAA</td></tr>
<tr><td>T9540C</td><td>COX3</td><td>L111L</td><td>A382</td><td>0</td><td>65</td><td>TACCCCCCAA[T|c]TAGGAGGGCA</td></tr>
<tr><td>G15301A</td><td>CYTB</td><td>L185L</td><td>A382</td><td>0</td><td>133</td><td>ACTTCATCTT[G|a]CCCTTCATTA</td></tr>
<tr><td>C16223T</td><td>D-loop</td><td></td><td>A382</td><td>0</td><td>168</td><td>GCAATCAACC[C|t]TCAACTATCA</td></tr>
<tr><td>T146C</td><td>D-loop</td><td></td><td>U20S</td><td>0.97</td><td>37</td><td>ATTCCTGCCT[T|c]ATCCTATTAT</td></tr>
<tr><td>G3010A</td><td>l-rRNA</td><td></td><td>U20S</td><td>1</td><td>87</td><td>AGGACATCCC[G|a]ATGGTGCAGC</td></tr>
<tr><td>C3699G</td><td>ND1</td><td>G131G</td><td>U20S</td><td>0.98</td><td>85</td><td>CCCTGATCGG[C|g]GCACTGCGAG</td></tr>
<tr><td>T4216C</td><td>ND1</td><td>Y303H</td><td>U20S</td><td>1</td><td>64</td><td>ACTTATATGA[T|c]ATGTCTCCAT</td></tr>
<tr><td>A10398G</td><td>ND3</td><td>T113A</td><td>U20S</td><td>1</td><td>35</td><td>ATTAGACTGA[A|g]CCGAATTGGT</td></tr>
<tr><td>G10685A</td><td>ND4L</td><td>A72A</td><td>U20S</td><td>1</td><td>98</td><td>GCGAAGCAGC[G|a]GTGGGCCTAG</td></tr>
<tr><td>A11251G</td><td>ND4</td><td>L164L</td><td>U20S</td><td>1</td><td>127</td><td>TCATCGCACT[A|g]ATTTACACTC</td></tr>
<tr><td>A12612G</td><td>ND5</td><td>V92V</td><td>U20S</td><td>1</td><td>62</td><td>TCATCCCTGT[A|g]GCATTGTTCG</td></tr>
<tr><td>T13281C</td><td>ND5</td><td>V315V</td><td>U20S</td><td>0.99</td><td>127</td><td>TCATAATAGT[T|c]ACAATCGGCA</td></tr>
<tr><td>G13708A</td><td>ND5</td><td>A457T</td><td>U20S</td><td>0.98</td><td>93</td><td>TAAACGCCTG[G|a]CAGCCGGAAG</td></tr>
<tr><td>A13933G</td><td>ND5</td><td>T532A</td><td>U20S</td><td>0.99</td><td>115</td><td>CCCTAGCATC[A|g]CACACCGCAC</td></tr>
<tr><td>T14798C</td><td>CYTB</td><td>F17L</td><td>U20S</td><td>1</td><td>61</td><td>TAACCACTCA[T|c]TCATCGACCT</td></tr>
<tr><td>C15263A</td><td>CYTB</td><td>P172T</td><td>U20S</td><td>0.17</td><td>129</td><td>AGTAGACAGT[C|a]CCACCCTCAC</td></tr>
<tr><td>C15452A</td><td>CYTB</td><td>L235I</td><td>U20S</td><td>0.97</td><td>135</td><td>ACTTCTCTTC[C|a]TTCTCTCCTT</td></tr>
<tr><td>C16069T</td><td>D-loop</td><td></td><td>U20S</td><td>1</td><td>78</td><td>AGTATTGACT[C|t]ACCCATCAAC</td></tr>
<tr><td>C16108T</td><td>D-loop</td><td></td><td>U20S</td><td>0.98</td><td>71</td><td>ACATTACTGC[C|t]AGCCACCATG</td></tr>
<tr><td>T16126C</td><td>D-loop</td><td></td><td>U20S</td><td>1</td><td>89</td><td>ATGAATATTG[T|c]ACGGTACCAT</td></tr>
</table>
